# Evaluation of the impact of alveolar bone graft surgery on the nasal cavity of individuals with cleft lip and palate

**DOI:** 10.1590/1678-7757-2024-0212

**Published:** 2024-11-22

**Authors:** Maryana Lourenço Bastos do Nascimento, Ivy Kiemle Trindade-Suedam, Natalia Bortotti Loureiro, Maria Noel Marzano-Rodrigues, Marcela Cristina Garnica Siqueira, Thiago Henrique dos Santos Antunes Albertassi, Sergio Henrique Kiemle Trindade

**Affiliations:** 1 Universidade de São Paulo Hospital de Reabilitação de Anomalias Craniofaciais Unidade de Estudos do Sono Bauru SP Brasil Universidade de São Paulo, Hospital de Reabilitação de Anomalias Craniofaciais, Unidade de Estudos do Sono, Laboratório de Fisiologia, Bauru, SP, Brasil.; 2 Universidade de São Paulo Hospital de Reabilitação de Anomalias Craniofaciais Bauru SP Brasil Universidade de São Paulo, Hospital de Reabilitação de Anomalias Craniofaciais, Seção de Otorrinolaringologia, Bauru, SP, Brasil.

**Keywords:** Cleft lip and palate, Internal nasal dimensions, Alveolar bone graft

## Abstract

Cleft lip and palate are the most common congenital malformations in the craniofacial region, occurring at a rate of 1:700 births in Brazil. These conditions lead to functional impacts on patients, such as changes in breathing, teeth, speech, chewing, swallowing and sucking. Treatment begins with primary surgeries, including lip and palate repair, which aim to reconstruct the soft tissues. Secondary alveolar bone grafting (SABG) reconstructs the bone defect in the cleft region, with the main goal of supplying bone tissue to the cleft region and restore the continuity of the alveolar process.

**Objective:**

To measure the changes in cross-sectional areas (CSAs) and nasal volume in patients and their impact on the nasal cavity (NC) in the two-month postoperative period (PO2M).

**Methodology:**

This study included 15 patients with complete unilateral cleft lip and palate (U/CLP) indicated for alveolar bone grafting (ABG). Cone beam computed tomography scans obtained prior to SABG and at PO2M were compared. Nasal volumes and CSAs were measured by marking the masks delimiting the nasal cavity on CT scans using Mimics™ software.

**Results:**

NC volumes (total, right and left sides) were statistically lower at PO2M in patients with left-sided UCLP. In right-sided UCLP, these volumes were only significant for the total NC and left NC. The CSAs of the internal nasal valve in both groups showed significantly lower values compared to the preoperative period (p≤0.05).

**Conclusion:**

In the short term, alveolar bone graft surgery reduces the volume of nasal cavities and the cross-sectional areas of the right and left internal nasal valve as a whole, not only the cleft area where the graft material was placed.

## Introduction

Cleft lip and palate (CL/P) are the most common congenital malformations of the craniofacial region, occurring in Brazil at a rate of 1 in 700 births.^1,2^ The rehabilitation of these patients requires a multidisciplinary approach with a specialized and integrated team and involves complex treatments to achieve satisfactory outcomes for both patients and the healthcare team.^3,4^

Secondary alveolar bone grafting (SABG) plays a crucial role in the rehabilitation process of patients with CL/P. This procedure stabilizes the maxillary bone structure, the upper dental arch, and periodontium, while also promoting the development of permanent teeth in the cleft region and enabling orthodontic movement. Additionally, it allows for the placement of endosseous implants, when necessary, closes oronasal fistulas, and provides bone support for the nasal alar base. Overall, SABG enhances the patient's aesthetic harmony by improving nasal symmetry.^5–9^

SABG using autologous iliac crest cancellous bone is a standard procedure for patients with CL/P during the mixed dentition phase. It addresses both functional and aesthetic outcomes, correlating with improvements in patient quality of life, satisfaction and high surgical success rates. During surgery, the nasal floor, which is invaginated into the cleft region and often associated with an oronasal fistula, is repositioned upwards and sutured, effectively reconstructing the nasal fossa anatomy. This procedure reshapes the nasal base and wing through by grafting bone tissue into the affected area.^10–12^

The presence of the cleft itself impairs the development of the nasomaxillary complex, causing a deviated septum, nostril atresia, and hypertrophy of the nasal turbinates on the contralateral side.^13^ These alterations reduce the internal dimensions of the nasal cavity (NC) and increase resistance to respiratory airflow, often leading to mouth breathing^13^, which can impact craniofacial development and compromise lower airway function.^14–20^

Considering the frequent complaints of nasal obstruction by patients in the early postoperative period of SABG, it is important to assess the impact of this procedure on internal nasal dimensions. Therefore, this study aimed to determine the effect of SABG surgery on nasal volume and the cross-sectional areas of the nasal valve in patients with respiratory complaints two months after the procedure. We hypothesized that SABG surgery reduces nasal cavity dimensions, potentially explaining these respiratory complaints.

## Methods

### Study group, sample size, design and settings

To determine whether patients’ complaints were directly related to the SABG procedure, which has been shown to alter nasal morphology in patients with complete unilateral cleft (UCL/P) (Lee, et al.^21^ (2013)), we conducted a comparative evaluation of the CBCT scans taken before and after surgery for clinical purposes. This was essential to assess any changes in the upper airway, particularly in the nostril region. The study protocol was approved by the Institutional Review Board (institution name and protocol number omitted for peer review).

A total of 15 patients were enrolled in the study, and their pre- and postoperative CBCT scans were selected to evaluate possible morphological changes in the airways of patients with complete UCL/P shortly after SABG. A convenience sampling method was used, in accordance with the eligibility criteria.

The study included both male and female patients aged 10 to 16 years with complete unilateral clefts undergoing routine orthodontic treatment, with indication for SABG. All participants were submitted to primary plastic surgeries in early childhood. Patients undergoing re-grafting, as well as those with genetic syndromes, other craniofacial anomalies or inflammatory diseases of the upper airway at the time of CBCT were excluded. All patients attended a 60-day postoperative follow-up and reported nasal complaints after surgery.

### SABG technique and CBCT characteristics

The SABG procedure was performed by a single oral and maxillofacial surgeon using the surgical technique described by Boyne and Sands^22^ (1972), which is widely used in our institution.^22,23^ The surgery involves making an oblique buccal incision reaching the central portion of the first molar and meeting an intra-sulcular incision extending up to the lateral cleft margin. The gingival boundary is then contoured, reaching the opposite segment of the maxilla and ending in the intra-sulcular region of the central incisors. From this incision, the buccal mucosa was divided, creating a full-thickness flap. The palatal mucosa was separated and sutured. The nasal floor mucosa was then repositioned superiorly and sutured to close fistulas, creating a space where the bone graft from the iliac crest was carefully placed. Finally, the buccal flap was repositioned over the bone graft. The entire divided bone extension was covered, and the incisions were sutured with simple stitches.^23^

Cone beam computed tomography scans were acquired using the i-CAT Next Generation CT scanner (ISI-iCAT Imaging System - cone beam, Next Generation i-CAT^®^), with the following specifications: field of view (FOV) of at least 13cm, allowing visualization of the upper airway, an exposure time of 26.9 seconds, 120 Kv, 37.07 mA and resolution of 0.25 voxels. The images were imported in DICOM (Digital Imaging and Communications in Medicine).^24–27^

### Morphometric analysis of the nasal cavity

To evaluate the NC volume, the Mimics™ software (Materialise, Belgium) was used. First, the CBCT scan files were opened, and a mask with a threshold range of −1024 to −500 Hounsfield units, consistent with air density, was created. This tool allows the NC to be filled in, distinguishing it from other structures such as soft and hard tissues. Using the coronal plane, a section was selected to provide the best visualization of thresholds for the creation of the initial mask, with the following anatomical boundaries: the anterior limit of the nasal valve, the posterior limit of the choanae, the lower and upper limits of the nasal floor, and the turbinates and middle meatus, related to the respiratory portion.

After creation of the first mask, areas not of interest, such as the paranasal sinuses, ethmoidal cells, and regions of artifact, were excluded from the tomographic images using the Multiple Slice Edit. Additional structures that were not initially selected were added, considering different tomographic planes, until the NC was filled by the mask according to the predefined limits. Once the necessary edits were made, the mask was converted into a 3D object. The final step involved polishing and removing any spicules, after which the 3D reconstruction of the NC was complete, allowing volume measurements to be obtained.

To find the CSAs of the internal nasal valve (cleft and non-cleft sides), a coronal section was used. The first section anterior to the inferior nasal turbinate was defined as the most posterior reference point. A polygon was then outlined over the internal nasal valve region, and the area was calculated. The creation of 3D models enabled the measurement of total nasal volumes, right NC volumes, left NC volumes, and the CSAs of internal nasal valves.

All reconstructions were performed by two trained and calibrated examiners, who obtained different nasal measurements. To assess intra- and interexaminer reproducibility, examiner 1 performed reconstructions on all 15 preoperative and 15 postoperative scans twice, while examiner 2 conducted reconstructions on 50% of the postoperative scans, also twice. Both examiners repeated their initial measurement after a 30-day interval (T1 and T2). The mean values from both examiners across the two measurements were used for statistical analysis. Intra- and interexaminer reproducibility for volumes and CSAs showed an intraexaminer ICC > 0.80 for both examiners, while the interexaminer ICC was 0.80, confirming the high reproducibility of measurements for both CSAs and volumes.

### Analysis of results

The Kolmogorov-Smirnov test was used to assess the normality of the data. For comparison of quantitative variables, data with normal distribution were presented as mean ± standard deviation and compared by the paired Student's t-test. A pvalue ≤ 0.05 was considered significant. The power to detect mean differences between pre- and postoperative total NC volumes and cross-sectional areas of internal nasal valves (R and L), at a 95% confidence interval was 44.67%.

## Results

### Sample characterization

The study sample included 15 individuals aged 10 to 16 years (13.00±1.96) with indication for SABG surgery and complete unilateral cleft lip and palate. Of the 15 participants, six had right-sided clefts and nine had left-sided clefts. The sample included 11 males (63.63%) and 4 females (36.36%).

### Volumes and cross-sectional areas

Table 1 summarizes the decrease in the nasal cavity volume and cross-sectional areas as a whole in the postoperative period for the 15 patients with unilateral clefts, both on the right and left sides.

**Table 1:** Pre- and postoperative nasal cavity (NC) volumes (mm^3^), and internal nasal valve (NV) cross-sectional areas (CSA) (mm^2^) of individuals with unilateral cleft lip and palate (N=15).

Variable	Preop (mean±sd)	Postop (mean±sd)	p-value (alpha≤0.05)
**NC Volume (mm^3^)**			
Total	15,194±3,724	13,409±3,362	<0.0001*
Right	9,219±2,668	8,354±2,690	0.0115*
Left	7,974±1,908	6,674±1,585	0.0024*
**NV CSA (mm^2^)**			
Right	106.20±34.79	90.78±32.90	<0.0001*
Left	102.50±28.48	84.61±28.48	0.0006*

The mean total cavity volume decreased from 15,194 mm³ preoperatively to 13,409 mm³ postoperatively (p≤0.0001). The volume of the right cavity decreased from 9,219 mm³ preoperatively to 8,354 mm³ postoperatively (p≤0.0115), while the left cavity volume decreased from 7,974 mm to 6674 mm³ (p≤0.0024). Thus, when the 15 patients were analyzed without separation by cleft laterality, there was a statistically significant decrease in total nasal volume, as well as in the volumes of both the right and left cavities.

The CSA of the right NV decreased from 106.20 mm^2^ to 90.78 mm^2^ after surgery, while the CSA of the left NV decreased from 102.50 mm^2^ to 84.61 mm^2^. There was a statistically significant reduction in the cross-sectional areas of the right and left nasal valves (p≤0.05).

The pre- and postoperative NC volumes (mm³) and CSAs of individuals with unilateral cleft lip and palate (UCL/P) were evaluated according to cleft laterality corresponding to the side where the grafted material was placed (Table 2). The analysis revealed that patients with left-sided UCL/P showed a significant decrease in total nasal cavity volume, from 14,583 mm^3^ to 12,712 mm^3^ after surgery (p≤0.001). On the side contralateral to the cleft, the volume decreased from 9,202 mm^3^ to 7,867 mm^3^ after surgery (p≤0.0001). On the left side, corresponding to the cleft side, the volume was reduced from 7,429 mm^3^ to 6,375 mm^3^ after the procedure (p≤0.0275). Additionally, the nasal valve CSAs on the cleft side decreased from 95.21 mm^2^ to 74.25 mm^2^ (p≤0.0020), and on the non-cleft side from 118.70mm^2^ to 101.20mm^2^ (p≤0.0020). All showed statistically significant differences.

**Table 2:** Pre and postoperative nasal cavity (NC) volumes (mm^3^), and internal nasal valve (NV) cross-sectional areas (CSA) (mm^2^) of individuals with right (n=6) and left (n=9) side unilateral cleft lip and palate (N=15).

Cleft side	Variable	NC Volume (mm³)	Preop (mean±sd)	Postop (mean±sd)	p-value (alpha≤0.05)
Right (n=6)	NC Volume (mm³)	Total	16,110±5257	14,456±4,556	0.0123*
		Cleft-side	9,244±3869	9,085±3,797	0.4041
		Non-cleft side	8,792±2438	7,121±1,652	0.0135*
	NV CSA (mm^2^)	Cleft-side	87.59±21.43	75.18±19.32	0.0312*
		Non-cleft side	113.40±33.36	99.03±31.28	0.0156*
Left (n=9)	NC Volume (mm^3^)	Total	14,583±2,438	12,712±2,332	0.0017*
		Cleft side	7,429±1,347	6,375±1,562	0.0275*
		Non-cleft side	9,202±1,762	7,867±1,729	<0.0001*
	NV CSA (mm^2^)	Cleft-side	95.21±23.98	74.25±23.05	0.0020*
		Non-cleft side	118.70±37.37	101.20±36.83	0.0020*

For patients with right-sided UCL/P, a reduction in total nasal cavity volume was also observed, with a preoperative volume of 16,110 cm^3^, which decreased to 14,456 cm^3^ postoperatively (p≤0.0123). The left contralateral cavity volume decreased from 8,792 mm^3^ to 7,121 mm^3^ after the procedure (p≤0.0135). However, on the right cleft side that received the graft, no statistically significant difference in volume was noted, with only a numerical decrease from 9,244 mm³ to 9,085 mm³ postoperatively (p≤0.4041). In terms of the cross-sectional areas of the internal nasal valve, the CSA on the cleft side decreased from 87.59 mm^2^ to 75.18 mm^2^ postoperatively (p≤0.0312). The CSA also decreased on the non-cleft side, from 113.40 mm^2^ to 99.03 mm^2^ (p≤0.0156).

**Figure 1:**
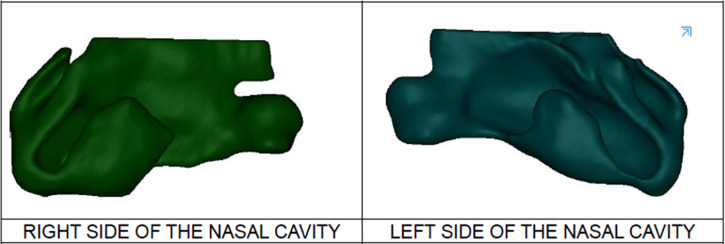
3D reconstructions of the total nasal cavity preoperatively in patients with UCLP. reconstructed by Mimics^®^ Software, Materialise, used to obtain measurements of total nasal volume, left and right cavity volume and the cross-sectional areas of the internal nasal valve on both sides.

**Figure 2:**
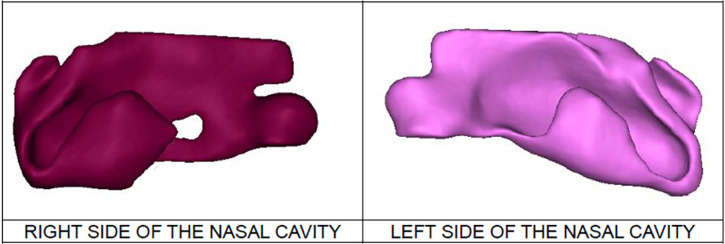
3D reconstructions of the total nasal cavity postoperatively in patients with UCLP, reconstructed by Mimics^®^ Software, Materialise, used to obtain measurements of total nasal volume, left and right cavity volume and the cross-sectional areas of the internal nasal valve on both sides.

Three-dimensional CAD models of the nasal cavities of all study participants are shown in Figure 1 preoperatively and Figure 2 postoperatively. Qualitatively, the postoperative nasal cavities were smaller, especially in the internal nasal valve.

Right is a 3D reconstruction of the right side of the nasal cavity of patient 1, used as an example. On the left, the left side of the nasal cavity.

On the right is a 3D reconstruction of the right side of the nasal cavity of patient 1, used as an example. On the left, the left side of the nasal cavity.

## Discussion

Patients in this study reported nasal obstruction after undergoing SABG surgery. Chang, et al.^28^ (2017) found that children who underwent SABG surgery were more likely to report nasal obstruction than those who did not. Similarly, Lee, et al.^21^ (2013) identified morphological changes at the base of the nostrils in children who underwent SABG. Our study aimed to examine the effects of SABG on NC volumes and CSAs in patients with UCL/P, seeking to elucidate the breathing difficulties reported by these patients. While a direct cause and effect cannot be confirmed, the morphological changes in the nasal cavity after SABG may be related to nasal obstruction, as other conditions linked to respiratory symptoms were ruled out.

Despite being able to reject the null hypothesis, the study results should be interpreted with caution due to certain limitations. The small, heterogeneous sample size and power values below 80% may limit generalizations (Crutzen and Peters, 2017; Serdar, et al.^29^ (2021)). However, as the sampling was based on patients with a common post-SABG complaint, altering these parameters might raise ethical concerns. Since this is a before-and-after study, comparisons within the same patient reduce possible biases. The absence of a validated respiratory symptoms questionnaire also represents a limitation.

Our study demonstrated a reduction in total NC volume in patients with UCL/P. For those with left-sided UCL/P, reductions were observed on both the cleft side and the contralateral side. In patients with right-sided CL/P, the volume decreased on the contralateral side, with no statistically significant reduction on the cleft side, likely due to a smaller sample size. Conversely, the reduction in the contralateral NC volume in rightsided CL/P suggests that SABG affects not only the cleft side. Reductions in the CSA of the internal nasal valve were seen in both right and left UCL/P patients, with the region closest to the graft site being the most affected. This suggests that SABG reduces the internal nasal dimensions, due to elevation of the floor into the nasal cavity, which was previously invaginated into the cleft area.

Additionally, CL/P can affect maxillary sinus development in the cleft side. Kiaee, et al.^30^ (2021) found that children with operated unilateral complete cleft, both with and without palatal fistula, had smaller maxillary sinuses on the fistula side. This reduction may increase the risk of sinus disease. Their study closely aligns with ours in terms of age group, cleft type, and choice of software to measure the volume of the tomographic images. Our findings point to a reduction in the total nasal cavity volume and the CSAs of the nasal valve regions, which could further reduce the maxillary sinus volume on the grafted side and constitute a risk factor for sinus diseases. Additionally, another study from Kiaee, et al.^31^ (2021) showed that the maxillary sinus volume was smaller on the cleft side, suggesting that surgeries in the nasal cavities and the maxillary sinus floor could impact both respiratory and sinusal physiology.

Sijmons, et al.^32^ (2023), in the Netherlands, also investigated the impact of alveolar bone grafting through tomographic analysis. While their findings differ from ours (showing no reduction in internal nasal dimensions a year after surgery), their study used a different software, only assessed total nasal cavity volume, and had a different postoperative timespan than ours. This supports our hypothesis that changes may be less significant in the long term.

The SABG is generally performed during mixed dentition, before the eruption of the permanent canine adjacent to the cleft. Thus, the procedure is performed on growing patients, which may induce changes in nasal volume Analyzing each nasal cavity individually must account for the "nasal cycle", a physiological process in which congestion and decongestion affect both sides.^33,34^ This means that the decrease in volume and cross-sectional areas of the nasal valve on the non-cleft side may not be related to anatomical causes, but rather result from the nasal cycle itself. However, differences between the cleft and non-cleft sides were consistently observed in most cases analyzed, as reported by Kunkel, Wahlman and Wagner^35,35^ (1997, 1999), who found a 35% smaller nasal volume on the cleft side.

Cone beam computed tomography is the gold standard for assessing upper airway morphology and internal geometry in various patient groups.^36–39^ As such, we used the Mimics Research^®^ software, which has good accuracy for reconstructing the nasal cavity and was already used in the research laboratory at (institution name omitted for peer review).^40–44^ This software offers the possibility of creating a mask only in the region of interest, and the Multiple Slice Edit tool allows easy and accurate exclusion of structures not suitable for the group, such as ethmoid cells and paranasal sinuses. If necessary, structures can also be added to certain sections that were not considered when creating the mask.

Comparing our results with previous studies is challenging due to limited research in this field. However, Kiaee, et al.^30,31^ (2021) highlighted smaller sinus dimensions on the cleft and palatal fistula sides, suggesting that ABG may reduce sinus volumes on the grafted side, thus increasing the risk of future sinus diseases. As such, patients and clinicians should be aware that sinusal symptoms may occur after surgery.

From a clinical point of view, aside from the possible higher risk of sinusitis, it should be noted that patients may experience transient symptoms of nasal obstruction shortly after surgery. Sijmons, et al.^32^ (2023) report that these symptoms tend to diminish over time.

Our study consistently showed a significant reduction in total nasal volume and CSAs within the first two months after surgery. Unilaterally, there was a decrease in patients with left CLP on both sides of the nasal cavity, and in patients with right CLP there was a statistically significant decrease on the side contralateral to the cleft. This supports our hypothesis that the internal nasal valve region is the area most affected by SABG in the short term. Further research, including our ongoing study on ABG's effects on sinus morphology, will help clarify its long-term impacts on nasal dimensions and symptoms.

## Conclusion

The analysis of internal nasal dimensions in patients with UCLP confirmed the hypothesis that SABG reduces nasal cavity dimensions, which would explain the respiratory complaints of patients. Alveolar bone grafting induces short-term morphophysiological changes, decreasing both the volume of the nasal cavities and the cross-sectional areas of the internal nasal valves on both sides, rather than only on the cleft area where the graft material was placed. These reductions in the internal dimensions of the nasal valve region may be linked to nasal obstruction symptoms shortly after surgery.

## Data Availability

All data generated or analyzed during this study are included in this published article
